# Supplementary material for: The Behaviours in Dementia Toolkit: A Descriptive Study on the Reach and Early Impact of a Digital Health Resource Library About Dementia-Related Mood and Behaviour Changes
Source: Geriatrics (Basel). 2025 Jun 11;10(3):79. doi: 10.3390/geriatrics10030079 (PMC12192763; doi:10.3390/geriatrics10030079)
Supplement: Supplementary file 1 [file geriatrics-10-00079-s001.zip › geriatrics-3621010-supplementary.pdf]

Supplementary Materials

Figure S1: Behaviours in Dementia Toolkit homepage and main navigation.

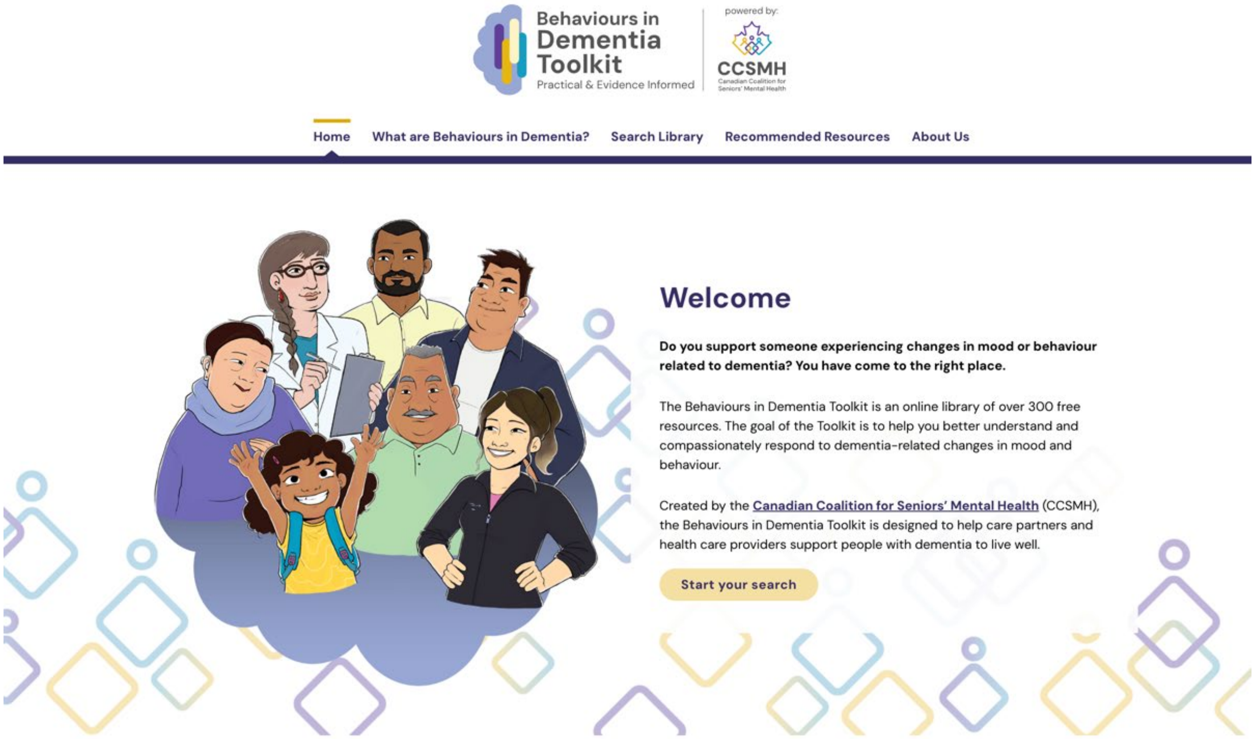

Figure S2: Behaviours in Dementia Toolkit search library function.

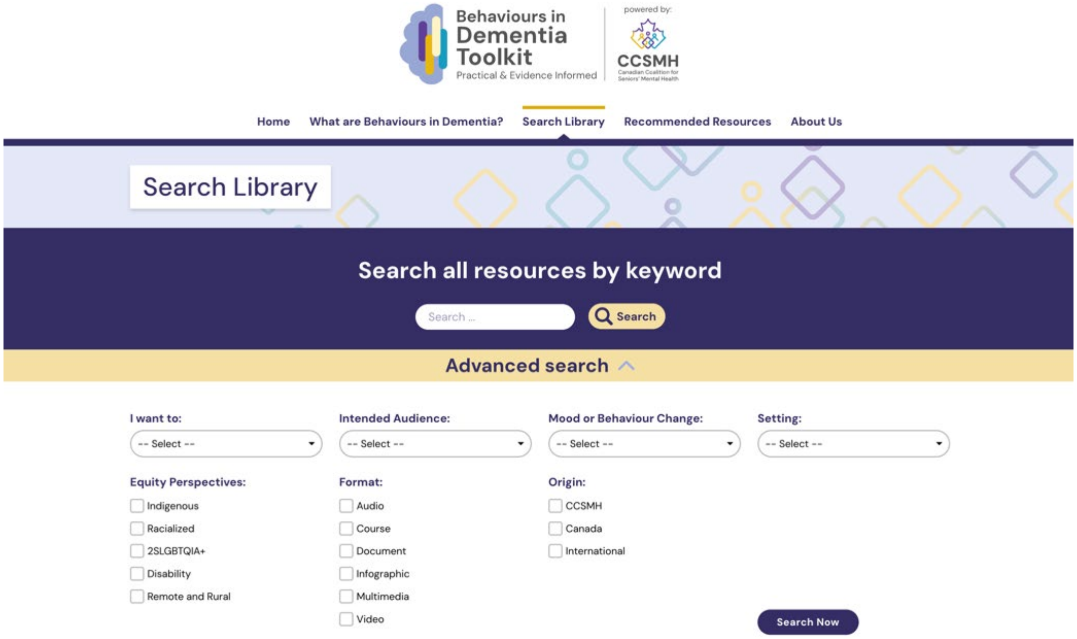

Figure S3: Behaviours in Dementia Toolkit search categories for informal and formal care partners

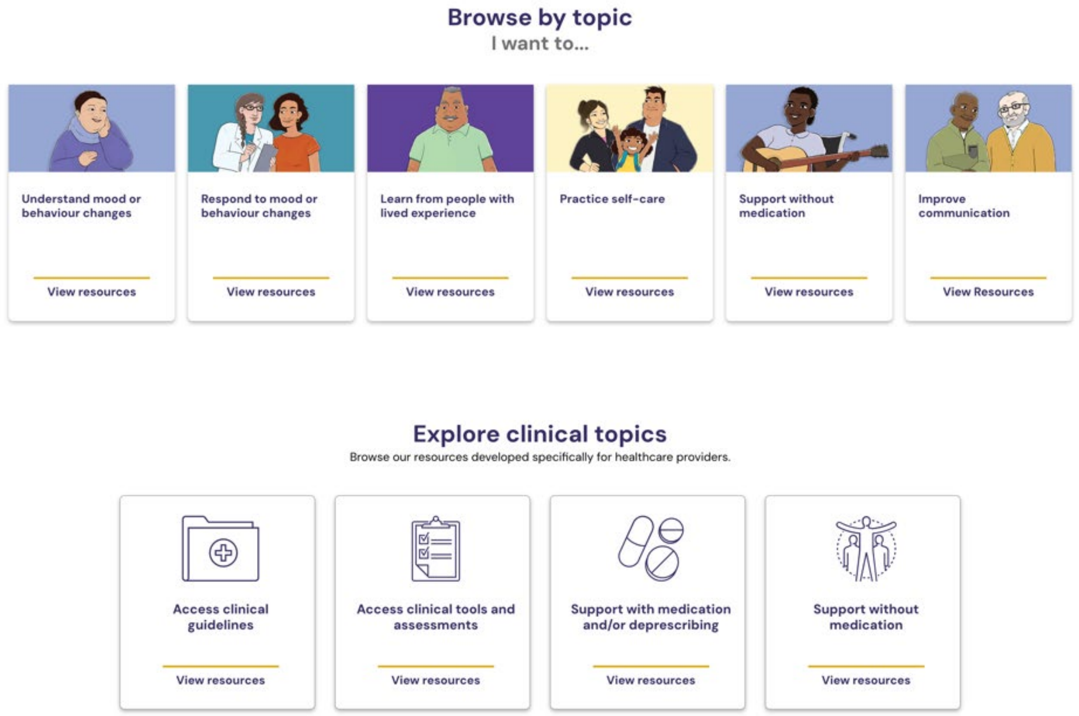

Table S1: Behaviours in Dementia Toolkit resource collection snapshot.

**Category**	
Tag	*Total resources*
Understand mood or behaviour changes	177
Respond to mood or behaviour changes	163
Improve communication	80
Learn from people with lived experience	49
Practice self-care	25
Support without medication	51
Access clinical guidelines	36
Access clinical tools and assessments	37
Support with medication and/or deprescribing	32
**Intended audience**	
Tag	*Total resources*
Public	235
Healthcare provider	134

**Mood or behaviour changes**	
Tag	*Total resources*
Anger	19
Desire to move	22
Eating habits	7
Fear or worry	25
Hygiene	11
Interest in daily activities	18
Repeating words or actions	9
Sadness	17
Sleep	16
Social interactions	13
Thoughts, beliefs, or perceptions	24
Other	18
**Equity perspectives**	
Tag	*Total resources*
Indigenous	14
Racialized	10
2SLGBTQIA+	16
Disability	9
Remote and rural	5
**Format**	
Tag	*Total resources*
Document	184
Audio	7
Video	81
Course	17
Infographic	34
Multimedia	7
**Origin**	
Tag	*Total resources*
CCSMH	45
Canada	236
International	78
**Setting**	
Tag	*Total resources*
Acute care	17

Assisted living	15
Support in community	37
Long-term care home	34

Table S2: Human-centered design inspiration phase activities for the Behaviours in Dementia Toolkit

**Inspiration phase activities (April to October 2023)**	**Activity description**
1. Working Group established	Multidisciplinary team met monthly for the duration of the project.
2. Survey of information needs and preferences of two primary audiences	Collected 285 surveys from key audiences including health care providers, care partners, etc. identifying their information needs, preferences, and suggestions related to behaviours in dementia.
3. Environmental scan to understand how older adults find health information	Conducted an environmental scan to examine the ways older adults find, receive, and share health information [16].
4. Environmental scan of existing resources	Conducted an international scan which identified 1408 English-language resources.
5. Collection statement and guidelines	Led by a librarian to guide the process of collection development.
6. Focus group with older adults/with lived experience	Five older adults with lived experience attended a session to scope and name the Toolkit.
7. One-to-one meetings with equity deserving groups	Met with organizations serving the following equity deserving groups including remote & rural; Indigenous; 2SLGBTQIA+; and Black perspectives on dementia care.
8. Persona-empathy mapping exercise	Completed six empathy maps of potential user 'personas' to identify knowledge gaps.
9. Card sort activity	Seven participants sorted sample resources into proposed categories and provided feedback on their suitability as navigation options.
10. Let's Talk Behaviours in Dementia webinar	145 people attended our webinar. In the Q&A and pre/post poll we learned about information needs of health care providers and care partners.

Table S3: Human-centered design ideation phase activities for the Behaviours in Dementia Toolkit.

**Ideation phase activities (August 2023 to January 2024)**	**Activity description**

11. Wireframe development	Worked with web design contractor to mock up website and library.
12. Custom metadata schema development	Developed a system of categorizing and naming to facilitate the search functions of the library and supply consistent clear language descriptions for each resource [24].
13. Content development	Wrote content for website and library pages, headers/footers, buttons, filters, etc.
14. Custom illustrated characters	Worked with illustrator contractor to create characters and illustrations for the Toolkit.
15. Indigenous Welcome to the website	Worked with Elder Larry Frost to develop an Indigenous Welcome for the homepage.
16. Selection & curation of resources	Led by a librarian. Selected 318 relevant, practical, evidence-informed, and inclusive resources to include in the Toolkit.
17. Narrative blog posts co-created with experts	Worked with 13 experts to co-develop experiential articles containing resource recommendations.
18. Informational videos developed	Created three informational videos to support website use and navigation.
19. Specialized infographics for different audiences developed	Developed 18 infographics curating resources for different audiences, settings, and types of dementia.

Table S4: Human-centered design implementation phase activities for the Behaviours in Dementia Toolkit.

**Implementation phase activities (November 2023 to March 2024)**	**Activity description**
20. Beta website development	Worked with web design contractor to develop a beta version of the website and library.
21. User satisfaction of beta site examined	Analyzed 12 individual interviews. This sub-study will be detailed in a separate publication [25].
22. Usability of beta site examined	Analyzed 12 individual interviews. This sub-study will be detailed in a separate publication currently in development.
23. Acceptability of content on beta site assessed	Analyzed 76 surveys. This sub-study will be detailed in a separate publication currently in development.
24. Metadata schema refined	Consulted with advocates, researchers, and people with lived experience to find a balance of accurate, clear, and inclusive terminology. This process is detailed in a separate publication [24].
25. Custom lay descriptions and descriptive metadata records	Led by a Library Technician and Librarian.

developed for each resource using metadata schema	
26. Website re-development	Worked with web design contractor to make changes based on usability study. Both functional and design changes were made. This sub-study will be detailed in a separate publication [25].
Outreach activities	Many strategies used to promote the Toolkit.
Reach of website post-launch assessed	See Methods and Results.
Impact of website post-launch assessed	See Methods and Results.

Table S5: Electronic survey questions

Survey question	Response options
1. What is your connection to dementia?	Select all that apply.<br>a. I am living with dementia.<br>b. I am caring for someone living with dementia.<br>c. I work in a clinical role supporting people living with dementia.<br>d. I work in a non-clinical role connected to dementia (e.g., educator, researcher, not-for-profit).<br>e. I know someone living with dementia.<br>f. I have a general interest in dementia.<br>g. Other (specify)
2. Where do you live?	a. Alberta<br>b. British Columbia<br>c. Manitoba<br>d. New Brunswick<br>e. Newfoundland and Labrador<br>f. Northwest Territories<br>g. Nova Scotia<br>h. Nunavut<br>i. Ontario<br>j. Prince Edward Island<br>k. Quebec<br>l. Saskatchewan<br>m. Outside of Canada<br>n. Prefer not to say
3. Why did you visit the Behaviours in Dementia Toolkit website?	Open text field

4. Rate your agreement with the following statements about the website.<br>I. I found the website unnecessarily complex.<br>II. I thought the website was easy to use.<br>III. I think that I would need the support of a technical person to be able to use this website.<br>IV. I found the various functions in this website were well integrated.<br>V. I thought there was too much inconsistency in this website.<br>VI. I would imagine that most people would learn to use this website very quickly.<br>VII. I found the website very cumbersome to use.<br>VIII. I felt very confident using the website.<br>IX. I needed to learn a lot of things before I could get going with this website.	Please select the best option for each statement.<br>a. strongly disagree<br>b. somewhat disagree<br>c. neither agree nor disagree<br>d. somewhat agree<br>e. strongly agree
5. How did the Behaviours in Dementia Toolkit impact your knowledge about behaviours in dementia?	Please select the best option.<br>a. I had no change in knowledge.<br>b. The Toolkit validated my current knowledge, beliefs, and activities.<br>c. The Toolkit increased my knowledge of the topic.
6. How did the Behaviours in Dementia Toolkit impact your ability to provide care to people experiencing behaviours in dementia?	Please select the best option.<br>a. I had no change in my ability to provide care.<br>b. The Toolkit validated my current approaches to care.<br>c. The Toolkit increased my ability to provide care.
7. Please rate your agreement with the following statements about the Behaviours in Dementia Toolkit.<br>I. The Behaviours in Dementia Toolkit is relevant to my situation.	Please select the best option for each statement.<br>a. strongly disagree<br>b. somewhat disagree<br>c. neither agree or disagree<br>d. somewhat agree

II. The Behaviours in Dementia Toolkit is feasible to use in my situation.<br>III. The Behaviours in Dementia Toolkit is something I intend to use.<br>IV. The Behaviours in Dementia Toolkit makes a meaningful contribution to improving access and use of dementia advice and support in Canada.<br>V. I would recommend the Behaviours in Dementia Toolkit to others to use.	e. strongly agree<br>f. I don’t know
